# Novel mutations in breast cancer patients from southwestern Colombia

**DOI:** 10.1590/1678-4685-GMB-2019-0359

**Published:** 2020-11-18

**Authors:** Melissa Solarte, Carolina Cortes-Urrea, Nelson Rivera Franco, Guillermo Barreto, Pedro A. Moreno

**Affiliations:** ^1^ Universidad del Valle, School of Systems and Computing Engineering, Bioinformatics and Biocomputing Laboratory, Cali, Colombia.; ^2^ Universidad del Valle, Biology Department, Human molecular Genetic Laboratory, Cali, Colombia.

**Keywords:** Breast cancer, tumor, genetics of cancer, SNP, exome sequencing

## Abstract

Breast cancer is the leading cause of death by cancer among women in less developed regions. In Colombia, few published studies have applied next-generation sequencing technologies to evaluate the genetic factors related to breast cancer. This study characterized the exome of three patients with breast cancer from southwestern Colombia to identify likely pathogenic or disease-related DNA sequence variants in tumor cells. For this, the exomes of three tumor tissue samples from patients with breast cancer were sequenced. The bioinformatics analysis identified two pathogenic variants in *Fgfr4* and *Nf1* genes, which are highly relevant for this type of cancer. Specifically, variant FGFR4-c.1162G>A predisposes individuals to a significantly accelerated progression of this pathology, while NF1-c.1915C>T negatively alters the encoded protein and should be further investigated to clarify the role of this variant in this neoplasia. Moreover, 27 novel likely pathogenic variants were found and 10 genes showed alterations of pathological interest. These results suggest that the novel variants reported here should be further studied to elucidate their role in breast cancer.

Breast cancer is a multifactorial disease that involves a complex combination of genetic and environmental factors. It is estimated that approximately 5% to 10% of disease cases are hereditary and related to mutations in DNA damage detection and repair genes, while approximately 90% of cases are sporadic with no family history of breast cancer (Oosterwijk *et al.*, 2014).

In Colombia, studies evaluating the genetic factors related to familial breast cancer report mutations that explain approximately 20% of cases (Briceño-Balcázar *et al.*, 2017). Therefore, research in this field should take advantage of high-throughput technologies, such as next-generation sequencing (NGS), to search for novel variants in additional genes involved in this pathology. This study characterized the exome of three patients with breast cancer from southwestern Colombia to identify likely pathogenic sequence variants in tumor cells that may be involved in this neoplasia.

The patients were recruited from Centro Medico Imbanaco (Imbanaco Medical Center) in the city of Cali, Colombia. All study participants provided written informed consent for genetic analysis. Participant consent and the study protocol were approved by the ethics committee of the Imbanaco Medical Center. The use of DNA samples for exome sequencing was authorized by the Ministry of Health and Social Protection, complying with all established protocols and standards. As inclusion criteria, patients should have a personal history of cancer diagnosis only related to breast cancer and could not have received hormone treatment, chemotherapy, or radiation therapy (Table 1).

**Table 1:** General clinical characteristics of the three patients with breast cancer from Southwest Colombia.

	Sample 1	Sample 2	Sample 3
Diagnosis	Invasive Ductal	Invasive Lobular	Invasive Ductal
Stage	IB	IA	IA
Age at diagnosis	68	66	66
Age at menarche	15	13	14
Number of children	1	3	NA
Number of births	1	3	NA
Number of abortions	0	0	NA
Family history of first- and second-degree relatives with cancer	Yes	Yes	Yes
Type of familial cancer	Breast, liver, lung	Breast, colon	Breast

Patient 1, from Cali, Colombia, was 68 years old and diagnosed with stage II invasive ductal carcinoma in the right breast. She had a family history of breast cancer (first-degree relatives, mother and two sisters), liver cancer (one brother), and lung cancer (another sister died at 80 years of age). She experienced menarche at 15 years of age and menopause at 51 years of age; had one child, one birth, and no abortions; breastfed for two months; and had her child before 30 years of age. The sample was obtained through a bilateral total mastectomy, with the right breast being removed for treatment and the left breast being removed for prophylactic or preventive reasons.

Patient 2, from Cali, Colombia, was 66 years old and diagnosed with stage II invasive lobular carcinoma in the right breast. She had a second-degree family history of breast cancer (paternal grandmother) and a first-degree history of colon cancer (father). She experienced menarche at 13 years of age and menopause at 52 years of age; had three children, three births, and no abortions; breastfed for 12 months; and had her first child before 30 years of age. The sample was obtained through a total mastectomy of the right breast.

Patient 3, from Cali, Colombia, was 66 years old and diagnosed with stage II invasive ductal breast cancer in the left breast. She had a first-degree family history of breast cancer (mother and sister). She experienced menarche at 14 years of age and menopause at 45 years of age and was nulliparous. The sample was obtained by thick needle biopsy (Tru-Cut) of the right breast.

A brief description of the three samples is provided in Table 1. The samples were sequenced at Macrogen sequencing center in Korea using the Agilent SureselectXT V5 PostCap Enrichment System for sequencing on an Illumina HiSeq 4000 system. The paired-end FASTQ files were aligned to the human reference genome GRCh37 (hg19) using BWA-MEM (Burrows-Wheeler Alignment Tool) (Li and Durbin, 2009). Duplication marking was performed with Picard tools. Putative variants were analyzed with the HaplotypeCaller Genome Analysis Toolkit (GATK v3.0) according to the best practices of the Broad Institute (McKenna *et al.*, 2010). In this study, only the single nucleotide polymorphism (SNP) variant call format (VCF) file was considered. For annotations based on databases, the latest versions of the following databases were used: dbSNP version 142 (Sherry *et al.*, 1999), ExAC version 0.3.1 (Karczewski *et al.*, 2017), and COSMIC version 70 (Forbes *et al.*, 2017). The first two databases contain population-wide variant information and COSMIC is the database for cancer variants. Additionally, functional annotations were performed to predict the possible effects of the variants at the protein level and evaluate if the variants are likely pathogenic. For this, PolyPhen-2 (Adzhubei *et al.*, 2010), SIFT (Kumar *et al.*, 2009), and SnpEff (Cingolani *et al.*, 2012) tools were used. In particular, for one variant, another functional predictor (Mutation Taster) was used to provide certainty for this annotation (Schwarz *et al.*, 2014) since only SnpEff generated a functional annotation for this variant.

A panel of 50 genes was constructed after extensively reviewing databases, such as Genecards (Stelzer *et al.*, 2016) and Genetics Home Reference (National Library of Medicine (US)). The panel comprised genes involved in the development of breast cancer tumors, including genes showing hereditary and somatic pathogenic mutations, in addition to diagnostic genes for breast cancer (invasive ductal and invasive lobular). We do not report the gene panel constructed here because only two alterations of clinical importance were found in the panel.

The following filters were applied to the variants according to the research interest of this study: i) All variants located in scaffolds but not chromosomes were excluded. ii) All non-exonic variants were eliminated, i.e., all functional variants in noncoding RNA, upstream and downstream DNA regions, intergenic and intronic regions, and 3’ and 5’ untranslated regions (UTRs). iii) Synonymous exonic variants were discarded.

The exonic variants located in the gene panel proposed here were identified and all likely pathogenic variants were considered. Furthermore, novel variants received particular interest and were proposed for future validation. For these variants, a literature review was carried out to determine which genes harboring these novel variants could be related to the neoplasm.

After analyzing the entire exome, novel likely pathogenic variants and known pathogenic variants were identified after filtering the gene panel. We proceeded to explore the functional pathways involving these genes and their proteins using the Kyoto Encyclopedia of Genes and Genomes (KEGG) database. For the three exomes, a total of 16727 exonic variants were found. Of these, 865 SNPs annotated as pathogenic were identified, including 27 that had not been reported for any population (Table S1).

In particular, one variant with a harmful effect was found in the *Fgfr4* gene (c.1162G>A; p.Gly388Arg). This mutation occurred only in the exome of patient 1 in the heterozygous state. In addition, a variant with an uncertain effect was found in the *Nf1* gene (c.1915C>T; p.*639Arg), causing a loss of the stop codon). This variant was found in the three exomes; specifically, in a heterozygous state for patients 1 and 2, while in a homozygous state for patient 3. For other genes in the panel, most variants found did not show clinical involvement in the neoplasm, i.e., synonymous mutations and missense mutations that were annotated and reported as benign.

The polymorphism c.1162G>A; p.Gly388Arg of the *Fgfr4* gene has been reported as pathogenic for cancer and tumor progression and is associated with a significant reduction in disease-free survival (Ulaganathan *et al.*, 2015). This variant was only present in the exome of patient 1 in a heterozygous state. According to the ExAC database, this variant presents a higher allelic frequency in Latin and East Asian populations, with values of 0.4337 and 0.4495, respectively.

The alteration c.1915C>T; p.*639Arg of the *Nf1* gene is a known nonstop variant. This type of variant leads to the loss of the stop codon and causes termination failure in normal translation and also likely results in persistent translation of downstream messenger RNA in the UTR 3’ (Inada and Aiba, 2005). This variant presents an allelic frequency of 0.5 in the Latin American population according to the ExAC database; furthermore, it is less frequent in Africa (0.2579) and more frequent in Europe (0.6695). This SNP was found in the three patients, specifically in the heterozygous state in patients 1 and 2 and in the homozygous state in patient 3. The possible effects of this variant have yet to be evaluated.

A comparative analysis of normal tissue with breast tumor tissue using the ONCOMINE database (Rhodes *et al.*, 2004) showed contradictory outcomes between different studies evaluating overexpression or underexpression of *Fgfr4* and *Nf1* genes. For example, some studies found significant differences in the overexpression of these genes, while others reported significant differences in the underexpression of *Fgfr4* and *Nf1* genes. Overall, there is no consensus on gene expression changes in breast tumor tissue versus normal tissue.

The functional pathways involving the variant-containing genes and their proteins were explored through PANTHER and KEGG functional databases. Many of these genes are involved in very different pathways, while only a few genes share close pathways. Particularly, *Ube4b* (Zhang *et al.*, 2014), *Arhgef4* (Cook *et al.*, 2014), *Gadd45b* (Fan *et al.*, 2017), *Arrb2* (Shenoy *et al.*, 2012), *Dtx1* (Boral *et al.*, 2017), *Timp1* (Lawicki *et al.*, 2016), *Cldn25* (Zhao *et al.*, 2015), *Sfn* (Shiba-Ishii and Noguchi, 2012), *Stab1* (Liu *et al.*, 2015) and *Dlec1* (Zhang *et al.*, 2015) genes are involved in key functional pathways (Figure 1), such as the cell cycle, motility, cellular adhesion, and important signal transduction pathways (MAPK, NOTCH, and HIF-1), among others. A malfunction in these pathways could trigger neoplasia, in this case, breast cancer. Likewise, some of these genes were explored in functional studies, which demonstrated that they are decisive in the prognosis, progression, and development of this pathology.

**Figure 1:**
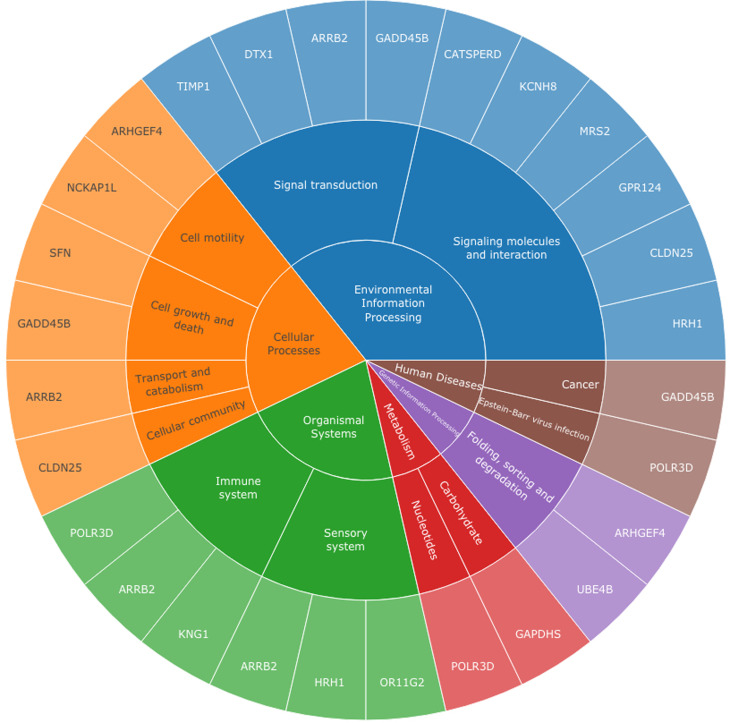
Functional pathways of genes in which variants were first reported.

The KEGG analysis and literature review showed that *Unc80*, *Zcchc2*, *Zkscan8*, *Gpr124*, *Hrh1*, *Kcnh8*, *Mrs2*, *Nckap1l*, *Or11g2*, *Tecpr2*, and *Tsc22d2* genes may not be related to this pathology given the functions in which they are involved. Accordingly, the variants in these genes are also not associated with breast cancer (Figure 1).

Overall, this study reports 27 novel and likely deleterious variants for Colombia and the world in 26 genes based on the evaluation of three exomes of breast cancer patients from southwestern Colombia. Among the 26 genes, *Dtx1*, *Ube4b*, *Timp1*, *Gadd45b*, *Arhgef4*, *Cldn25*, *Dlec1*, *Stab1*, *Sfn*, and *Arbb2* seem to be related to this type of cancer according to the functional pathway analysis, although a strong association with breast cancer is not reported for these genes. Moreover, 10 new mutations were found that could affect the functions of proteins encoded by key genes in breast cancer. Finally, FGFR4-c.1162G>A (exome 1) and NF1-c.1915C>T (exomes 1, 2, and 3) variants were also found, which confer a negative impact on this pathology.

## Supplementary material

The following online material is available for this article:

Table S1 Variants observed in the present study, reported for the first time in the world, with a pathogenic effect.
