# Supplementary material for: Novel mutations in breast cancer patients from southwestern Colombia
Source: Genet Mol Biol. 2020 Nov 18;43(4):e20190359. doi: 10.1590/1678-4685-GMB-2019-0359 (PMC7684693; doi:10.1590/1678-4685-GMB-2019-0359)
Supplement: Supplementary file 1 [file 1415-4757-GMB-43-4-e20190359-suppl1.pdf]

Genetics and Molecular Biology

https://doi.org/10.1590/1678-4685-GMB-2019-0359

Supplementary Material to "Novel mutations in breast cancer patients from southwestern Colombia."

Table S1 - Variants observed in the present study, reported for the first time in the world, with a pathogenic effect.

Chromosome	Position	Variant	SIFT		Polyphen2		SnpEff	Gene	Rs	Nucleotide Change	Aminoacid Change	Sample 1	Sample 2	Sample 3
chr1	10238703	Non synonymous	Deleterious	0.000	Probably damaging	0.99	Moderate	UBE4B	NA	c.3527G>C	p.Arg1176Thr	0|0	0|0	0|1
chr1	27189876	Non synonymous	Deleterious	0.001	Probably damaging	0.99	Moderate	SFN	NA	c.173C>A	p.Ala58Asp	0|0	0|0	0|1
chr2	131673843	Non synonymous	Deleterious	0.003	Probably damaging	0.96	Moderate	ARHGEF4	NA	c.1334C>A	p.Pro445His	0|0	0|0	0|1
chr2	210640695	Non synonymous	Deleterious	0.000	Probably damaging	0.99	Moderate	UNC80	NA	c.224G>T	p.Trp75Leu	0|1	0|0	0|0
chr3	11301202	Non synonymous	Deleterious	0.001	Probably damaging	1.0	Moderate	HRH1	rs1308725859	c.479T>A	p.Ile160Asn	0|1	0|0	0|0
chr3	19574908	Non synonymous	Deleterious	0.012	Probably damaging	0.99	Moderate	KCNH8	NA	c.2641G>A	p.Val881Ile	0|0	0|0	0|1
chr3	38087080	Non synonymous	Deleterious	0.000	Probably damaging	1.0	Moderate	DLEC1	NA	c.458A>G	p.Glu153Gly	0|1	0|0	0|0
chr3	52558177	Non synonymous	Deleterious	0.000	Probably damaging	0.99	Moderate	STAB1	NA	c.7604T>G	p.Val2535Gly	0|1	0|0	0|0
chr3	150176275	Non synonymous	Deleterious	0.001	Probably damaging	0.99	Moderate	TSC22D2	NA	c.2195T>G	p.Leu732Arg	0|1	0|0	0|0
chr3	186445036	Non synonymous	Deleterious	0.000	Probably damaging	1.0	Moderate	KNG1	rs777023083	c.575G>A	p.Gly192Glu	0|0	0|0	0|1
chr6	24423181	Non synonymous	Deleterious	0.000	Probably damaging	0.99	Moderate	MRS2	NA	c.1133G>T	p.Trp378Leu	0|0	0|0	0|1
chr6	24423182	Non synonymous	Deleterious	0.000	Probably damaging	1.0	Moderate	MRS2	NA	c.1134G>T	p.Trp378Cys	0|0	0|0	0|1
chr6	28121245	Non synonymous	Deleterious	0.000	Probably damaging	1.0	Moderate	ZKSCAN8	NA	c.1187A>G	p.His396Arg	0|0	0|0	0|1
chrX	47442905	Non synonymous	Deleterious	0.000	Probably damaging	1.0	Moderate	TIMP1	NA	c.91C>A	p.Pro31Thr	0|1	0|0	0|0
chr8	22103032	Non synonymous	Deleterious	0.017	Probably damaging	0.988	Moderate	POLR3D	rs1006397319	c.70C>T	p.Arg24Trp	0|0	0|1	0|0
chr8	37695299	Non synonymous	Deleterious	0.012	Probably damaging	0.994	Moderate	GPR124	NA	c.2101C>T	p.His701Tyr	0|0	0|0	0|1
chr11	66287095	Non synonymous	Deleterious	0.000	Probably damaging	0.999	Moderate	CTD-3074O7.11	NA	c.710T>C	p.Ile237Thr	0|1	0|0	0|0

Chromosome	Position	Variant	SIFT		Polyphen2		SnpEff	Gene	Rs	Nucleotide Change	Aminoacid Change	Sample 1	Sample 2	Sample 3
chr11	113650640	Non synonymous	Deleterious	0.039	Probably damaging	1.0	Moderate	CLDN25	rs17115958	c.123C>A	p.Asn41Lys	0|0	0|1	0|0
chr12	54891587	Non synonymous	Deleterious	0.013	Probably damaging	0.997	Moderate	NCKAP1L	NA	c.14C>G	p.Ser5Cys	0|0	0|0	0|1
chr12	113532893	Non synonymous	Deleterious	0.000	Probably damaging	0.997	Moderate	DTX1	NA	c.1433A>T	p.Lys478Met	0|1	0|0	0|0
chr14	20666158	Non synonymous	Deleterious	0.029	Probably damaging	0.968	Moderate	OR11G2	NA	c.664C>A	p.Leu222Ile	0|0	0|1	0|0
chr14	102906856	Non synonymous	Deleterious	0.000	Probably damaging	1.0	Moderate	TECPR2	NA	c.2662T>A	p.Tyr888Asn	0|0	0|1	0|0
chr17	4620555	Non synonymous	Deleterious	0.021	Probably damaging	0.97	Moderate	ARRB2	NA	c.464C>T	p.Pro155Leu	0|1	0|0	0|0
chr18	60242031	Non synonymous	Deleterious	0.015	Probably damaging	0.997	Moderate	ZCCHC2	NA	c.2717G>A	p.Gly906Asp	0|0	0|1	0|0
chr19	2477594	Non synonymous	Deleterious	0.000	Probably damaging	1.0	Moderate	GADD45B	NA	c.478C>T	p.Arg160Cys	0|0	0|0	0|1
chr19	5766114	Non synonymous	Deleterious	0.001	Probably damaging	0.985	Moderate	CATSPERD	NA	c.1507T>C	p.Ser503Pro	0|0	0|1	0|0
chr19	36033411	Non synonymous	Deleterious	0.003	Probably damaging	1.0	Moderate	GAPDHS	NA	c.560C>T	p.Ala187Val	0|1	0|0	0|0
